# The core mechanism of traditional Chinese medicine in improving myocardial fibrosis: restoring the dynamic balance of mitochondrial quality control

**DOI:** 10.3389/fcvm.2026.1970000

**Published:** 2026-09-29

**Authors:** Minxin Li, Ping Ni, Haijun Ma, Tianao Chen, Jin Dai, Muqi Jiao, Huan Zhang, Yingzhu Duan, Jian Liang, You Yu, Rui Yu

**Affiliations:** 1 Liaoning University of Traditional Chinese Medicine, Shenyang, Liaoning, China; 2 Liaoning University of Traditional Chinese Medicine Affiliated Second Hospital, Shenyang, Liaoning, China

**Keywords:** Chinese herbal monomers and formulas, mitochondrial autophagy, mitochondrial biogenesis, mitochondrial dynamics, mitochondrial protein proteostasis, mitochondrial quality control, myocardial fibrosis, traditional Chinese medicine

## Abstract

Myocardial fibrosis (MF), characterized by excessive collagen deposition, represents an inevitable pathological progression toward end-stage heart disease, underscoring the urgent clinical need for effective reversal strategies. In recent years, the mitochondrial quality control (MQC) system has emerged as a central regulatory hub for maintaining cardiac homeostasis, offering novel insights into the prevention and treatment of MF. This review bridges classical Traditional Chinese Medicine (TCM) therapeutic concepts with modern MQC mechanisms, primarily aiming to evaluate the intervention targets and efficacy of TCM monomers and formulas by detecting alterations in mitochondrial-associated protein expression, gene transcription levels, and organelle ultrastructure. By systematically reviewing experimental evidence of TCM-mediated MQC modulation, this paper highlights the multi-target and systematic advantages of TCM in combating MF, thereby injecting new momentum into the development of contemporary anti-MF therapeutics.

## 1 Introduction

Fibrosis can affect any organ and accounts for nearly half of all deaths (1). Previous studies have shown that approximately one-third of individuals with the four major cardiometabolic conditions—hypertension, type 2 diabetes, dyslipidemia, and obesity—have myocardial fibrosis (MF), and this proportion is expected to continue rising (2). Furthermore, as the population ages, the incidence of MF is gradually increasing. The serious consequences associated with MF—including heart failure, arrhythmias, and even death—pose a significant threat to public health (3).

MF is a pathological process triggered by cardiac injuries such as myocardial infarction, characterized by abnormal scar tissue formation, which leads to adverse ventricular remodeling. During this process, a large number of cardiac fibroblasts (CFs) are activated and differentiate into myofibroblasts. These myofibroblasts proliferate abnormally and extensively secrete collagen fibers and extracellular matrix (ECM), gradually replacing normal myocardial tissue (4). These alterations cause the originally pliable and synchronously contracting myocardium to become stiff and less compliant, resulting in decreased pumping efficiency and systolic dysfunction, ultimately driving the progression of heart failure. This process is accompanied by cardiomyocyte apoptosis and necrosis. It not only reflects the structural remodeling of myocardial tissue but also marks the transition of the disease from reversible injury to irreversible functional impairment, representing a core pathological manifestation shared by advanced stages of various cardiovascular diseases (5, 6). Currently, the specific mechanisms that contribute to its pathogenesis remain unclear. The primary goal of treatment is to slow the progression of MF rather than to reverse established fibrosis. Additionally, only a few therapies have demonstrated effectiveness or received clinical approval. As a result, modern medical treatment has significant limitations, and the associated costs can be relatively high (7–13). In contrast, traditional Chinese medicine (TCM) approaches diagnosis and treatment from a holistic perspective, emphasizing the harmony between heaven and humanity. It follows the principle of syndrome differentiation and treatment, utilizing multi-targeted and multi-modal interventions to address disease. Additionally, TCM is known for its mild adverse reactions and low treatment costs, providing patients with a viable alternative treatment option (14). As a result, TCM plays a crucial role in the treatment and prognosis of MF.

Research indicates that the onset and progression of MF are closely associated with mitochondrial dysfunction in cardiomyocytes (15). Mitochondria function as the energy powerhouses of the cell, generating adenosine triphosphate (ATP) through oxidative phosphorylation (OXPHOS). Additionally, they serve as a central hub regulating various biological processes, including cell signaling, metabolic balance, and apoptosis, which are essential for sustaining the cell's vital functions (16, 17). When mitochondrial function is impaired, the redox balance within the mitochondria becomes disrupted, which induces oxidative stress. This disruption leads to the release of damage-associated molecular patterns (DAMPs), including mitochondrial DNA (mtDNA), mitochondrial RNA (mtRNA), and reactive oxygen species (ROS). These factors trigger downstream inflammatory signaling pathways (18–20) and result in the abnormal opening of the mitochondrial permeability transition pore (mPTP). Consequently, this process leads to the release of apoptotic factors, such as cytochrome c (CYCS), into the cytoplasm, thereby activating downstream apoptotic pathways and impairing the cell's ability to survive and proliferate (21). Moreover, if mitochondrial protein import, folding, and maturation are abnormal, homeostasis is disrupted, and function is compromised; this directly affects cardiac function and can lead to associated myocardial diseases (22). Furthermore, mitochondrial dysfunction can also affect the tricarboxylic acid cycle (TCA cycle) and the functional electron transport chain (ETC), thereby disrupting tissue regeneration processes, including the differentiation and proliferation of cardiomyocytes (23, 24). Consequently, mitochondria act as a vital link that integrates nutrition, energy, and signaling. Their proper function is essential for cell survival and plays a key role in both cellular health and disease (25). To maintain this function, cells have evolved a sophisticated mitochondrial quality control (MQC) system, which dynamically maintains mitochondrial quality balance through processes such as biogenesis, dynamics, proteolysis, and autophagy (26, 27). During MF and cardiac remodeling, MQC—an essential endogenous regulatory system—helps cells adjust their energy metabolism and maintain cellular homeostasis by promoting adaptive changes in mitochondrial morphology and function. However, when MQC is disrupted, mitochondrial dysfunction worsens, initiating a cascade of adverse biological processes that ultimately contribute to the development of cardiac injury and MF (28). Therefore, maintaining the balance of the MQC is of great significance for the stability of cardiac structure and function.

A systematic literature search was conducted in the PubMed database to identify relevant studies. The search strategy combined the following keywords: “myocardial fibrosis,” “myocardial injury,” “mitochondrial quality control,” “mitochondrial biogenesis,” “mitophagy,” “mitochondrial dynamics,” “mitochondrial proteostasis,” “herbal monomers,” “herbal formulas,” and “traditional Chinese medicine,” using Boolean operators (AND) to combine these terms. The search was restricted to articles published between 2000 and 2026, and was supplemented by manual screening of reference lists to ensure comprehensive coverage of recent advances in this field. Original research studies (both *in vivo* animal models and *in vitro* experiments) were included if they met the following criteria: (1) investigated myocardial fibrosis or myocardial injury; (2) evaluated therapeutic interventions using TCM monomers or herbal formulas; and (3) simultaneously assessed fibrosis outcomes and at least one mitochondrial quality control (MQC)-related parameter. Studies were excluded if they were reviews, conference abstracts, case reports, non-TCM studies, or lacked MQC or fibrosis data. This structured and transparent search methodology enhances the reproducibility and methodological rigor of the present systematic review (Figure 1).

**Figure 1:**
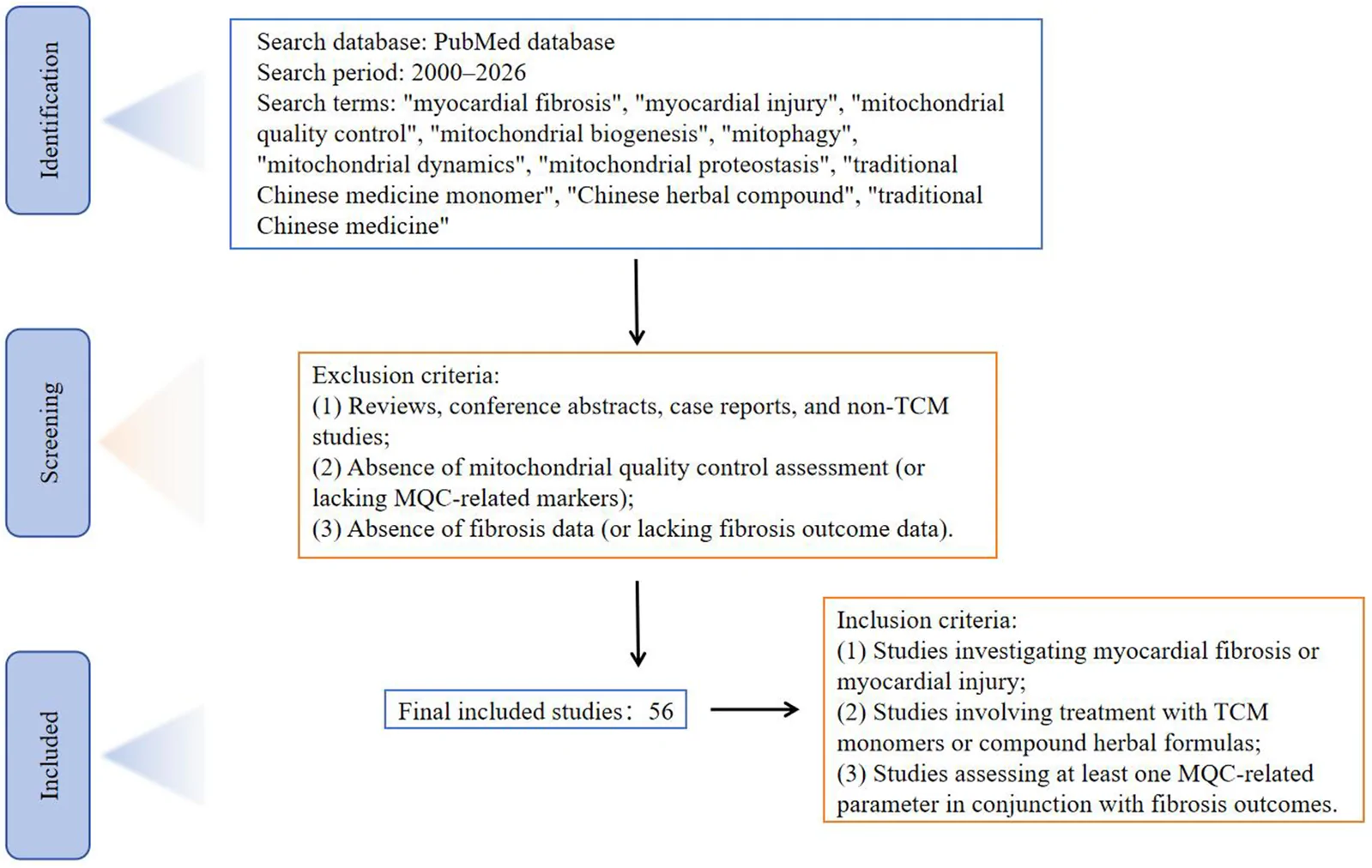
PRISMA flow diagram illustrating the literature search and screening process. The PubMed database was searched for studies published between 2000 and 2026 using keywords related to myocardial fibrosis, myocardial injury, mitochondrial quality control, and traditional Chinese medicine. After applying the exclusion and inclusion criteria, 56 studies were finally included in this review.

Upon reviewing the included literature, we found that many conclusions were drawn based on changes in mitochondrial protein expression. We further stratified the included studies by evidence strength. (i) “Protein/correlative evidence” refers to studies primarily reporting altered expression of MQC-related proteins (e.g., PGC-1α, TFAM, LC3/p62, PINK1/Parkin, Drp1/Mfn1/OPA1, or proteostasis/stress markers) together with fibrotic outcomes. (ii) “Functional readouts” indicate experiments that directly quantify mitochondrial function or mitophagy activity beyond static markers (e.g., mitochondrial membrane potential, mitochondrial ROS, mtDNA release, or mitophagy flux assays). (iii) “Causal evidence” requires active perturbation of MQC key nodes using gain- or loss-of-function approaches (genetic knockdown/knockout or overexpression, ideally with rescue experiments) and demonstration that both MQC functional outcomes and fibrosis-related phenotypes are directionally dependent on the manipulation. In the present review, most studies provided protein/correlative evidence, whereas only a subset further incorporated functional assays and/or causal genetic manipulation to substantiate MQC involvement. This evidence stratification is intended to clarify the current mechanistic strength and highlight experimental gaps for future MQC-directed research in myocardial fibrosis.

Based on the aforementioned literature screening and evidence evaluation, this article aims to systematically review current research progress on traditional Chinese medicine in the context of MF through the lens of MQC, grounded in TCM theory, to offer insights for developing novel therapeutic strategies against MF (Table 1).

**Table 1:** Mitochondrial quality control pathways involved in myocardial fibrosis and related experimental models.

MQC/mitochondrial pathway	Main cell type	*In vitro*/*in vivo*	Model/species/cell type	Induction method	Intervention/dose	Main fibrotic or cardiac endpoints
Mitochondrial biogenesis: SIRT1/PGC-1α/TFAM/NRF1	Cardiomyocytes, cardiac tissue	*In vivo*	dTGR rats, Sprague–Dawley rats, C57BL/6J mice, KKAy mice; neonatal rat cardiomyocytes	Ang II, pressure/metabolic stress, cardiac remodeling	Resveratrol	↑PGC-1α, ↑TFAM, ↑NRF1, ↑SIRT1; ↓ANP; improved mitochondrial biogenesis and remodeling
Mitochondrial biogenesis: AMPK/PGC-1α	Cardiomyocytes	*In vivo*/*in vitro*	H9c2 cells; experimental HF models	Cardiac injury/metabolic stress	Cinnamaldehyde	Improved heart failure, reduced MF, corrected energy metabolism
Mitochondrial biogenesis: AMPK/SIRT1/PGC-1α/NRF1/2	Cardiomyocytes, heart tissue	*In vivo*/*in vitro*	C57BL/6J mice; H9c2 cells; primary ventricular myocytes	Cardiac stress/injury	Salidroside	↑PGC-1α, ↑OXPHOS, ↑NRF1/2; ↓ROS; stabilized mitochondrial membrane potential
Mitochondrial biogenesis: ALDH2/HADH/MAOB	Cardiomyocytes	*In vivo*/*in vitro*	C57BL/6J mice; HL-1 cells; neonatal SD rat cardiomyocytes	Isoproterenol (ISO)	Formononetin	↓TGF-β1, ↓COL1A1, ↓COL3A1, ↓α-SMA; reduced MF
Mitochondrial biogenesis: APLN/SIRT3/PGC-1α	Heart tissue, cardiomyocytes	*In vivo*	db/db mice, db/+ controls; primary ventricular cardiomyocytes	Diabetic cardiomyopathy	Icariin	↑PGC-1α, ↑Mfn2, ↓collagen accumulation, improved mitochondrial function
Mitochondrial biogenesis: PPAR-γ/TGF-β/Smad2/3	Cardiac fibroblasts	*In vivo*	Spontaneously hypertensive rats (SHRs), WKY rats; rat CFs	Hypertension-related remodeling	Curcumin	↓CTGF, ↓PAI-1, ↓COL3, ↓FN; suppressed fibroblast activation
Mitochondrial biogenesis: BTG2/AMPK-FoxO3a	Heart tissue	*In vivo*	Doxorubicin cardiotoxicity model	Doxorubicin	Linggui Zhugan Decoction	Improved mitochondrial injury, restored energy metabolism
Mitochondrial biogenesis: PGC-1α/ATP-ADP balance	Heart tissue	*In vivo*	Heart failure model	Heart failure/remodeling	Xinfuli granule	↑PGC-1α, ↑ATP/ADP ratio, improved mitochondrial structure
Mitochondrial biogenesis: AMPK/PGC-1α/NRF1/mtTFA	Heart tissue	*In vivo*	Chronic heart failure model	Heart failure	Danggui Buxue Decoction	↑ATP, ↑ATP5D, improved fiber organization, ↓collagen accumulation
Mitophagy: PINK1/Parkin/NDP52	Cardiomyocytes	*In vitro*	H9c2 cells, HEK293T cells	Radiation injury	Aloe-emodin	↓mtROS, corrected LC3/p62/PINK1/Parkin abnormalities
Mitophagy: SIRT6/AMPK/FoxO3a	Heart tissue	*In vivo*	C57BL/6J mice	Chronic intermittent hypoxia	Berberine	↑PINK1, ↑Parkin; ↓COL1A1, ↓COL3A1, ↓TGF-β, ↓α-SMA
Mitophagy: JNK/c-Jun/miR-221	Heart tissue	*In vivo*	Sprague–Dawley rats	Diabetes-induced injury	Luteolin	↓p62, ↑LC3-II/LC3-I; enhanced autophagy, reduced MF
Mitophagy: miR-223-3p/FoxO3/ATG7	Cardiac fibroblasts	*In vitro*	Wistar rat CFs, 293 T cells	High glucose/fibrotic stimulus	Quercetin	Inhibited CF proliferation; regulated LC3-II/I and p62
Mitophagy: AMPK/mTOR	Heart tissue	*In vivo*	C57BL/6J mice	Doxorubicin cardiotoxicity	Ginsenoside Rb1	↓p62, ↓LC3-II, ↑NRF2; reduced cardiac injury
Mitophagy: SIRT3/FoxO1/BNIP3/LC3B	Heart tissue	*In vivo*	MI-associated chronic HF models	Myocardial infarction	Shenfuyixin granules	↑SIRT3, ↑BNIP3, ↑LC3B-II; reduced apoptosis
Mitophagy: miR-27a-3p/AMPKα2/PINK1-Parkin	Heart tissue	*In vivo*	Heart failure model	Pathologic remodeling	Huangqi-Danshen decoction	Restored mitophagy, improved cardiac function
Mitophagy: PINK1/Parkin/LC3/p62	Heart tissue	*In vivo*	Rat model of advanced HF	Doxorubicin	Yang-xin-xue keli	↑ATP, ↓LC3B, reduced myocardial loss and fibrosis
Mitophagy: PINK1/Parkin	Heart tissue	*In vivo*	Chronic HF after MI	Myocardial infarction	Nuanxinkang	Improved cardiac function, promoted mitophagy
Mitophagy: PI3K/Akt/mTOR	Heart tissue	*In vivo*	Reactive myocardial fibrosis model	Heart failure/remodeling	QiShenYiQi pill	↑Beclin1, ↑LC3B, ↓p62; activated autophagy
Mitophagy: NR4A1	Heart tissue/cardiomyocytes	*In vivo*	Advanced HF rats	Heart failure	Xin Fu Kang oral liquid	Corrected excessive mitophagy, improved mitochondrial homeostasis
Mitophagy: PINK1/Parkin/Beclin1/p62	Heart tissue	*In vivo*	Joint fatigue + AMI mice	Acute myocardial infarction with fatigue	Sheng Mai Yin	Reduced fibrosis, improved hypoxia tolerance and cardiac function
Mitophagy: PINK1/Parkin	Heart tissue	*In vivo*	Joint fatigue + myocardial injury rats	Myocardial injury	Huangqi Shengmai Decoction	Reduced pathological injury and fibrosis
Mitochondrial dynamics: Drp1/Mfn1/2/OPA1	Cardiomyocytes	*In vivo*/*in vitro*	C57BL/6J mice; NRCMs; H9c2 cells	Diabetic cardiomyopathy	Rhein	↓p-Drp1 S616/Drp1, ↑Mfn1/2, ↑OPA1; reduced apoptosis
Mitochondrial dynamics: PKCε/Stat3/Mfn2	Cardiomyocytes, heart tissue	*In vivo*	Sprague–Dawley rats; primary cardiomyocytes	Doxorubicin	Paeonol	↑Mfn2, improved fusion, reduced cardiotoxicity
Mitochondrial dynamics: GRP78/CHOP	Cardiomyocytes	*In vivo*/*in vitro*	BALB/c mice; HL-1 cells	Isoproterenol	Baicalein	↓Drp1, ↓MFF, reduced ER stress and MF
Mitochondrial dynamics: FoxO3a/Mfn1	Heart tissue, cardiomyocytes, CFs	*In vivo*/*in vitro*	C57BL/6J mice; NRCMs; NRCFs; H9c2 cells	Pathological remodeling	Breviscapine	↑Mfn1, ↓mtROS, improved fibrotic remodeling
Mitochondrial dynamics: PTP1B/Stat3/OPA1	Heart tissue, cardiomyocytes	*In vivo*/*in vitro*	Sprague–Dawley rats; primary cardiomyocytes	Diabetic cardiomyopathy	Punicalagin	↑OPA1, improved fusion, reduced fibrosis
Mitochondrial dynamics: Mfn2/OPA1/Drp1/Fis1	Heart tissue	*In vivo*	Ischemic HF model	Ischemia-induced injury	Astragali Radix–Notoginseng Radix et Rhizoma pair	Restored mitochondrial dynamic balance, reduced remodeling
Mitochondrial dynamics: Mfn1/Mfn2/OPA1	Cardiomyocytes	*In vitro*	Doxorubicin-damaged H9c2 cells	Doxorubicin	Active components of Sini decoction	Improved mitochondrial energetics and reduced MF
Mitochondrial dynamics: MCU/MARCHF5/MTFP1/Drp1	Heart tissue	*In vivo*	Ischemic HF model	Ischemia	QSYQ	Reduced excessive fission, improved energy metabolism
Mitochondrial dynamics: Mfn2/OPA1/Drp1/Fis1	Heart tissue	*In vivo*	Diabetic mice	Diabetes-related remodeling	Fufang Zhenzhu Tiaozhi	Corrected mitochondrial dynamics, reduced MF
Mitochondrial dynamics: PINK1/Parkin/Drp1/Fis1/MFF	Heart tissue	*In vivo*	Ischemic HF model	Ischemia	Ginseng Dingzhi Decoction	Promoted mitochondrial phagocytosis, inhibited fragmentation
Mitochondrial dynamics + mitophagy	Heart tissue	*In vivo*	Heart failure model	HF	Qishentaohong granules	Balanced fission/mitophagy, improved collagen deposition
Mitochondrial dynamics: NR3C1/Mfn2/cGAS-STING	Heart tissue	*In vivo*	Chronic HF mice	HF	Xin-Ji-Er-Kang	↓mtDNA release, ↓inflammation, ↓fibrosis
Mitochondrial dynamics: SIRT3/OPA1/Drp1	Heart tissue	*In vivo*	HF mice	HF	Xin-Ji-Er-Kang	Improved fusion/fission balance, reduced remodeling
Mitochondrial dynamics: Drp1/Fis1/TGF-β1/NF-*κ*B	Heart tissue	*In vivo*	Acute MI with circadian disruption	Myocardial infarction	Guanxin V	Reduced ventricular remodeling and fibrosis
Mitochondrial dynamics: RORα/p-Drp1/Mfn2/OPA1/PINK1-Parkin	Heart tissue	*In vivo*	Pressure overload + insomnia model	Pressure overload	Yiqi Huoxue Formula	Reduced collagen deposition, improved mitochondrial balance
Proteostasis/stress response: p38/p53/HSP70	Cardiomyocytes	*In vitro*	H9c2 cells	High glucose	Naringin	↓apoptosis, ↑MMP, ↑HSP27/HSP70
Proteostasis/ferroptosis-related: p53/SLC7A11/GPX4	Heart tissue, cardiomyocytes	*In vivo*/*in vitro*	C57BL/6J mice; H9c2 cells	Diabetes-related injury	Schisandrol B	Reduced oxidative stress and cardiac injury
Proteostasis/inflammatory fibrosis: MD2/TLR4	Heart tissue, iPSC-derived cells, 3D-EHTs	*In vivo*/*in vitro*	C57BL/6J mice; iPSC-CFs; iPSC-CMs; human primary fibroblasts; iPSC-ECs	Cardiac fibrosis	Artesunate	↓COL1A1, ↓ACTA2, ↓CTGF, ↓POSTN, ↓FN1; suppressed fibrotic gene program
Proteostasis/ER stress: GRP78/CHOP/caspase	Heart tissue	*In vivo*	Wistar rats	Myocardial injury/remodeling	Berberine	Reduced ER stress, apoptosis, and fibrosis

## 2 MQC and MF

There is a direct causal relationship between MF and MQC dysregulation. The MQC system maintains energy metabolism and redox balance in cardiomyocytes by coordinating mitochondrial biogenesis, dynamics, autophagy, and proteostasis. Once MQC function is impaired, it leads to mitochondrial structural damage and dysfunction, triggering excessive production of ROS and ATP depletion; this metabolic dysregulation further activates pro-fibrotic signaling pathways such as TGF-β/Smad, promotes abnormal activation of cardiac fibroblasts, and drives excessive secretion of the ECM, ultimately accelerating the pathological progression of MF (Figure 2).

**Figure 2:**
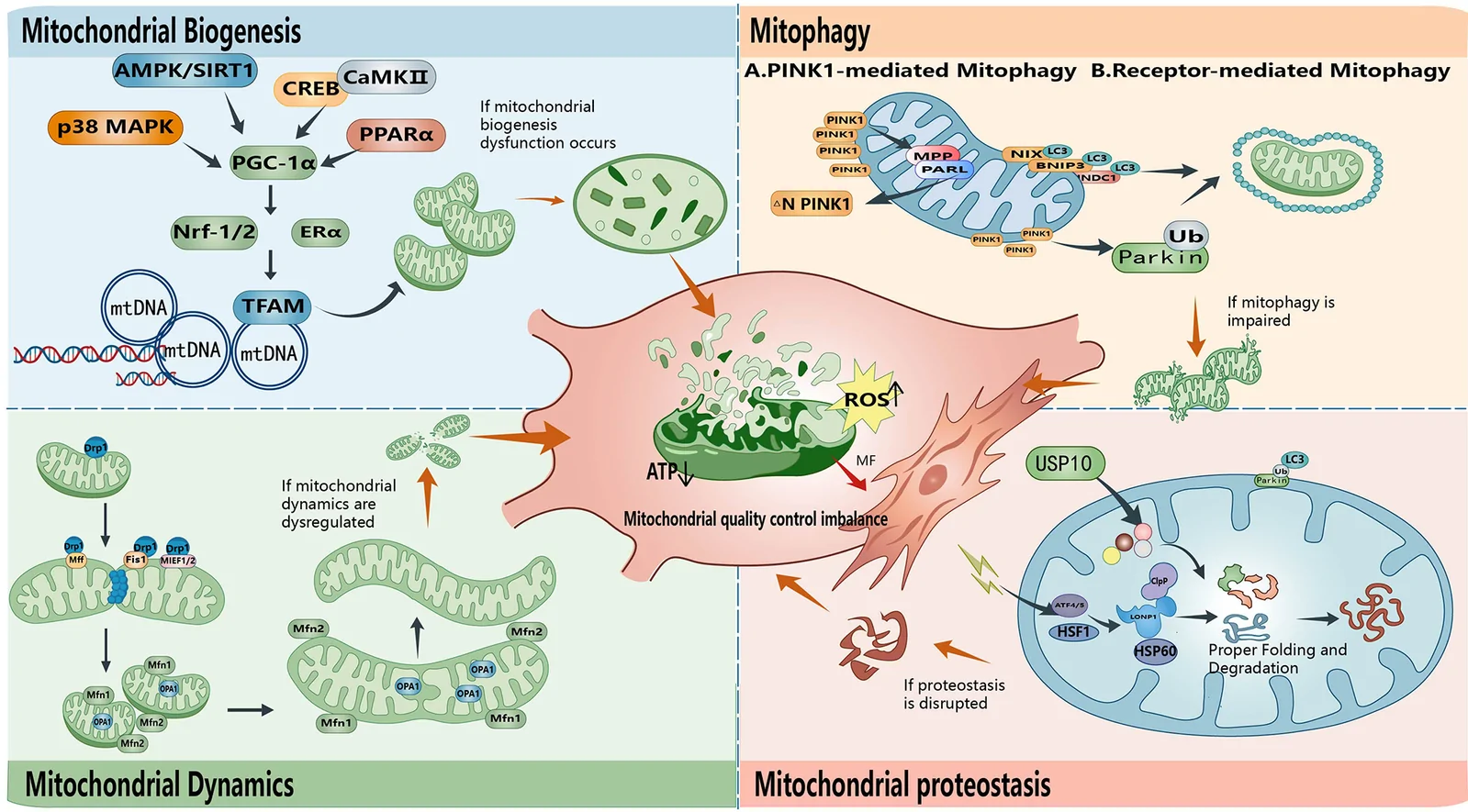
Core mechanism of myocardial fibrosis induced by mitochondrial quality control imbalance. This schematic illustrates the core pathological mechanism by which Mitochondrial Quality Control imbalance drives myocardial fibrosis. The MQC system comprises four key processes: mitochondrial biogenesis, dynamics, autophagy, and proteostasis. Dysfunction in any of these processes can lead to MQC imbalance, resulting in excessive ROS generation and insufficient ATP production. This triggers sustained oxidative stress and an energy crisis. Such metabolic dysregulation activates downstream pro-fibrotic pathways, such as TGF-β/Smad, ultimately promoting abnormal activation, proliferation, and excessive extracellular matrix secretion by cardiac fibroblasts, leading to myocardial fibrosis.

### 2.1 Mitochondrial biogenesis dysregulation and MF

Mitochondrial biogenesis is a finely coordinated process that relies on the coordinated transcription and replication of nuclear DNA (nDNA) and mtDNA (29). Through this process, existing mitochondria grow, divide, and renew themselves, which helps maintain their quantity and quality, while continuously supplying ATP to the cell (30). Mitochondrial biogenesis is regulated by the crucial factor peroxisome proliferator-activated receptor gamma coactivator-1alpha (PGC-1α). The activity of PGC-1α is influenced by phosphorylation from the upstream AMP-activated protein kinase (AMPK) and deacetylation by sirtuin 1 (SIRT1). Once activated, PGC-1α further stimulates nuclear respiratory factors 1 and 2, as well as the estrogen-related receptor α (ERRα), which promotes the expression of mitochondrial transcription factor A (TFAM). TFAM binds to mtDNA, initiating replication and transcription, ultimately promoting the generation of new mitochondria and completing the process of mitochondrial biogenesis (31). Mitochondrial biosynthesis also involves the calcium/calmodulin-dependent kinase II (CaMKII) pathway, which similarly acts on the cAMP response element-binding protein/PGC-1α axis, phosphorylating PGC-1α, enhancing its ability to bind to transcription factors, and promoting mitochondrial biogenesis; furthermore, p38 mitogen-activated protein kinase phosphorylates and activates PGC-1α, transporting it from the cytoplasm to the nucleus prior to the increase in transcripts of genes encoding mitochondrial proteins (32). These also include multiple pathways, such as the peroxisome proliferator-activated receptor α/PGC-1α pathway. Mitochondrial biological processes play a significant role in regulating cellular homeostasis (33). In a wild-type mouse model of MF induced by angiotensin II, researchers observed reduced levels of PGC-1α protein expression; upregulating its expression promoted mitochondrial biogenesis, improved mitochondrial function, and consequently inhibited the process of cardiomyocyte apoptosis. Notably, wild-type mice with cardiomyocyte-specific knockout of PGC-1α develop spontaneous MF even in the absence of angiotensin II intervention (34). Guo et al. (35), demonstrated that a deficiency in cardiomyocyte-specific neuraminidase 1 increases the expression of SIRT1, PGC-1α, and TFAM; this improves mitochondrial metabolism and alleviates oxidative stress, thereby mitigating MF. Further research has demonstrated that miR-34a directly downregulates its target gene SIRT1, accelerating the aging of vascular endothelial and smooth muscle cells and promoting the development of arterial inflammation and age-related cardiovascular diseases (36). Conversely, *in vivo* inhibition of miR-34a activates the SIRT1/PGC-1α/fibronectin type III domain protein 5 signaling pathway, downregulating markers of MF such as collagen I (COL1), collagen III (COL3), and transforming growth factor β1 (TGF-β1), thereby exerting an anti-MF effect (37). Research by Xu et al. (38), indicates that cinnamaldehyde (CIN) can significantly improve heart failure by reversing abnormal changes in markers of myocardial injury, inhibiting myocardial hypertrophy, and reducing MF, both *in vivo* and *in vitro*. Furthermore, CIN promotes myocardial fatty acid metabolism by activating the AMPK/PGC-1α signaling pathway, thereby correcting energy metabolism disorders.

### 2.2 Imbalance in the regulation of mitochondrial autophagy and MF

Mitochondrial autophagy serves as a key defense mechanism by which the heart resists damage, preserves function, and maintains cellular homeostasis through the selective clearance of dysfunctional mitochondria (39). Its initiation depends on the stable accumulation of PTEN-induced kinase 1 (PINK1) on the outer mitochondrial membrane, thereby triggering downstream ubiquitin-dependent autophagy signaling (40). Under physiological conditions, the PINK1 precursor protein enters healthy mitochondria, where it is sequentially cleaved by mitochondrial processing peptidases and PINK1/phosphoglycerate mutase family member 5-related diamidase-like enzymes. It is then returned to the cytoplasm and continuously degraded via the N-terminal regulatory pathway (41, 42). Upon mitochondrial depolarization, the degradation of PINK1 is inhibited, leading to its stable accumulation at the outer mitochondrial membrane. This process involves the phosphorylation of the Ser65 site on Parkin and ubiquitin molecules, which triggers the ubiquitination of various damaged mitochondrial proteins. Additionally, misfolded mitochondrial proteins may be handled by the pre-translational translocation-associated complex, initiating PINK1/Parkin-dependent mitochondrial autophagy (43). B-cell lymphoma-2 interacting protein 3 (BNIP3), FUN14 domain-containing protein 1 (FUNDC1), NIP3-like protein X, and microtubule-associated protein light chain 3 (LC3) bind to and are expressed on the outer membrane, representing a novel pathway for mitochondrial autophagy (44, 45). The development of MF is closely associated with both ubiquitin-dependent and non-ubiquitin-dependent pathways of mitochondrial autophagy. Moderate mitochondrial autophagy helps maintain the balance between mitochondrial quantity and quality, thereby enhancing mitochondrial function. However, when mitochondrial autophagy is insufficient or dysregulated, the accumulation of damaged mitochondria can trigger cell death, exacerbate cardiac dysfunction, and lead to cardiac remodeling and impaired contractile function (46). Studies have shown that in a model of doxorubicin (Dox)-induced cardiac injury, abnormal activation of tumor protein 53 (p53) can interact with Parkin, thereby interfering with its localization to dysfunctional mitochondria, inhibiting the initiation of mitophagy, exacerbating mitochondrial dysfunction, and promoting the development of MF (47). Experiments have also demonstrated that FUNDC1 deficiency can promote cell death by upregulating the expression of the lipid peroxidase acyl-CoA synthetase long-chain family member 4, thereby exacerbating lipid peroxidation and ultimately leading to MF (48). Fucoxanthin can downregulate the expression of key fibrotic factors such as TGF-β1, fibronectin (FN), and α-smooth muscle actin (α-SMA) by activating the BNIP3/Nix-mediated mitophagy pathway and the NRF2 signaling pathway, thereby alleviating oxidative stress and inhibiting the progression of MF (49). A research team led by Joseph C. Wu at Stanford University has proposed a novel synergistic therapeutic strategy for MF. This study simultaneously targeted the mechanosensitive protein SRC kinase in CFs and inhibited the TGF-β signaling pathway, thereby inducing the reversal of activated CFs to a quiescent state and achieving a “dual-pathway synergistic blockade” effect (50). This approach provides conceptual validation for the clinical treatment of MF, demonstrating a therapeutic strategy based on dual intervention targeting both mechanical and biochemical signals.

### 2.3 Abnormalities in mitochondrial dynamics (fusion/fission) and MF

Mitochondrial dynamics constitute a fundamental mechanism for maintaining cellular homeostasis; this dynamic equilibrium determines the morphology and function of the organelle by regulating mitochondrial fusion and fission. Mitochondrial fission, as a key component of this process, is the mechanism by which mitochondria proliferate. It is mediated by proteins such as dynamin-related protein 1 (Drp1), which, upon binding to fission protein 1 (Fis1), mitochondrial fission factor (MFF), and mitochondrial elongation factors 1 and 2, is recruited to the outer mitochondrial membrane, where it oligomerizes, forming a helical structure that encircles and contracts the mitochondrial membrane, thereby driving mitochondrial fission and facilitating mitochondrial proliferation (51–53). Mitochondrial fusion is regulated synergistically by mitofusin 1/2 (Mfn1/2) and optic atrophy protein 1 (OPA1); Mfn1/2 drives the fusion of the outer membranes of adjacent mitochondria via its GTPase activity (54–57), and OPA1-mediated inner membrane fusion and the functional diversity of its isoforms jointly regulate the structural and functional homeostasis of mitochondrial cristae. Overexpression of Mfn1/2 enhances mitochondrial fusion and inhibits apoptosis (58, 59), while downregulation of OPA1 induces spontaneous apoptosis (60, 61). Mitochondrial fission and fusion are dynamically and precisely regulated processes that effectively eliminate dysfunctional components, maintain mitochondrial homeostasis, and ensure normal functional output (53). Excessive fragmentation or impaired fusion can induce myocardial damage and mitochondrial dysfunction. Conversely, inhibiting the abnormal overexpression of proteins such as Drp1 can increase mitochondrial survival rates, thereby protecting the myocardium (28, 62, 63). Studies have found that increased expression of the piezo-type mechanosensitive ion channel component 1 promotes calcium ion influx in cardiomyocytes, activates calpain activity, and initiates the extracellular signal-regulated kinase 1/2 (ERK1/2) signaling pathway. This, in turn, induces Drp1 phosphorylation, driving mitochondrial fission and dysfunction. Conversely, deficiency of the piezo-type mechanosensitive ion channel component 1 can improve cardiac function and reduce fibrosis by inhibiting the ERK/Drp1 pathway and decreasing mitochondrial fission (64). Wu et al. (65), systematically elucidated, using various methods, that deficiency in mitochondrial phenylalanyl-tRNA synthetase 2 interferes with mitochondrial protein synthesis, induces mitochondrial fragmentation, and leads to dysregulated dynamics. At the cellular level, this manifests as upregulated expression of p-Drp1 S616 and downregulated expression of p-Drp1 S637, Mfn1, and Mfn2, triggering MF and increasing the risk of sudden death. This also indicates that pharmacologically inhibiting excessive mitochondrial fission or promoting mitochondrial fusion can effectively alleviate mitochondrial homeostasis imbalance and maintain the structural and functional integrity of cardiomyocytes, offering potential therapeutic value for treating related cardiomyopathies by modulating mitochondrial dynamics. Therefore, ensuring the normal operation of mitochondrial dynamics could serve as a novel therapeutic approach for preventing and treating MF.

### 2.4 Mitochondrial protein homeostasis disruption and MF

The proper functioning of mitochondria is highly dependent on their internal protein homeostasis, that is, the balance between protein synthesis, folding, modification, and degradation processes. It is worth noting that the majority of mitochondrial proteins are localized in the matrix, where their homeostasis is primarily maintained by the ATP-dependent protease system, including Lon peptidase homologues (Lon Peptidase 1, LONP1) and caseinopeptidase P. Among these, LONP1 is responsible for degrading misfolded proteins and regulating metabolism; caseinopeptidase P, on the other hand, must form a ClpXP complex with AAA+ chaperones (such as ClpX) to perform similar degradation functions. The two work in concert to clear abnormal proteins, constituting the fundamental quality control network within the mitochondria (50, 66, 67). Concurrently, molecular chaperone systems mediated by heat shock protein 60 and others assist in the correct folding and assembly of nascent polypeptide chains, thereby facilitating protein refolding (68, 69). When mitochondrial protein stress occurs, the pressure of protein folding increases, activating the mitochondrial unfolded protein response (UPR^mt^). This mechanism subsequently transmits stress signals to the cell nucleus, activating heat shock factor 1 and activating transcription factors 4/5 (ATF4/5) and other key transcription factors, inducing the upregulation of chaperone and protease expression. This system enhances the mitochondria's protein folding and degradation capacity, promotes the clearance of misfolded proteins, and restores mitochondrial protein homeostasis (22, 70). In addition to the UPR^mt^, two major proteolytic systems—the ubiquitin-proteasome system (UPS) and the autophagy-lysosome system—are also crucial for maintaining mitochondrial protein homeostasis (71, 72). The UPS marks misfolded, damaged, or redundant proteins for degradation by the proteasome through ubiquitination; the autophagy-lysosome system is responsible for the degradation of long-lived proteins, insoluble protein aggregates, cytoplasmic components, and even organelles. The two systems complement each other in the clearance of abnormal proteins, jointly maintaining intracellular protein homeostasis (73–75). Studies have demonstrated that the UPS is crucial in the development of MF. It primarily affects the function of cardiomyocytes and CFs by regulating the degradation of specific proteins, which in turn impacts the progression of MF (76). Liu et al. (77), demonstrated that targeting Sentrin-specific protease 1 to modulate the SUMOylation of heat shock protein 90α family class B members promotes their degradation; this finding lays the theoretical foundation for the development of novel intervention strategies for ischemic MF. Furthermore, other studies have found that sustained stress overload can induce elevated levels of serum heat shock protein 70 (HSP70) and promote its translocation to the myocardial cell membrane and interstitial regions. Antagonizing extracellular HSP70 significantly alleviates stress-induced myocardial hypertrophy and fibrosis, the mechanism may be related to the inhibition of HSP70/Toll-like receptor 4 (TLR4) signaling pathway activation, thereby reducing macrophage infiltration and the release of inflammatory cytokines (78). Furthermore, functional inhibition of extracellular HSP70 can downregulate the activation levels of the ERK and p38 signaling pathways, thereby improving hypertension-related myocardial hypertrophy and tissue remodeling (79). The atypical member of the HSP70 family, heat shock protein 12A, can serve as a scaffold protein to enhance the deubiquitination of p53 by ubiquitin-specific processing peptidase 10 (USP10), thereby inhibiting glycolysis and subsequently blocking the activation and fibrotic progression of cardiac fibroblasts (80). Another research team found that the upregulation of HSP47 can promote the deubiquitination of Sma mothers against decapentaplegic homologue 4 (Smad4) by recruiting USP10, thus activating the TGF-β1/Smad4 signaling pathway. Specific knockout of USP10 in CFs can eliminate the pro-fibrotic effects mediated by HSP47, providing a potential intervention strategy for fibrotic remodeling (81). Ding et al. (82), demonstrated that USP10 exerts its deubiquitinase activity through the His-679 site within its UCH domain, removing the K11/K48 ubiquitin chains from Mfn2 to prevent its degradation via the proteasomal pathway. This action maintains Mfn2 protein stability and mitochondrial functional homeostasis, thereby inhibiting cardiac hypertrophy and fibrosis. Specific deletion of the LONP1 protease in cardiomyocytes leads to the accumulation of abnormal proteins within mitochondria and impaired assembly of respiratory chain complexes, resulting in myocardial damage and inducing MF. Conversely, enhancing LONP1 expression can protect the heart by mitigating oxidative damage, maintaining redox homeostasis, and reducing the toxic effects of ROS (78, 83). Research by the Yang team (84) demonstrated that apoptosis-stimulating protein of p53 1 promotes the ubiquitination-mediated degradation of p53 by inhibiting the binding of the deubiquitinase OTUB1 to p53. Conversely, inhibition of ASPP1 reduces the activation of CFs and alleviates MF. The Sun (85) team discovered that BRG1-associated factor 155 can enhance the poly-ADP-ribosylation levels of its downstream targets by inhibiting the ubiquitination-mediated degradation of poly(ADP-ribose) polymerase 1 by E3 ubiquitin ligase 2, which contains a WW domain, thereby promoting the hypertensive-associated MF process.

## 3 TCM treats MF by regulating MQC

MF is a common pathological alteration in the progression of various cardiovascular diseases to their end stages. Existing studies have confirmed that TCM can effectively intervene in MF by modulating MQC to maintain its functional homeostasis, thereby providing a new direction for clinical prevention and treatment (Figure 3).

**Figure 3:**
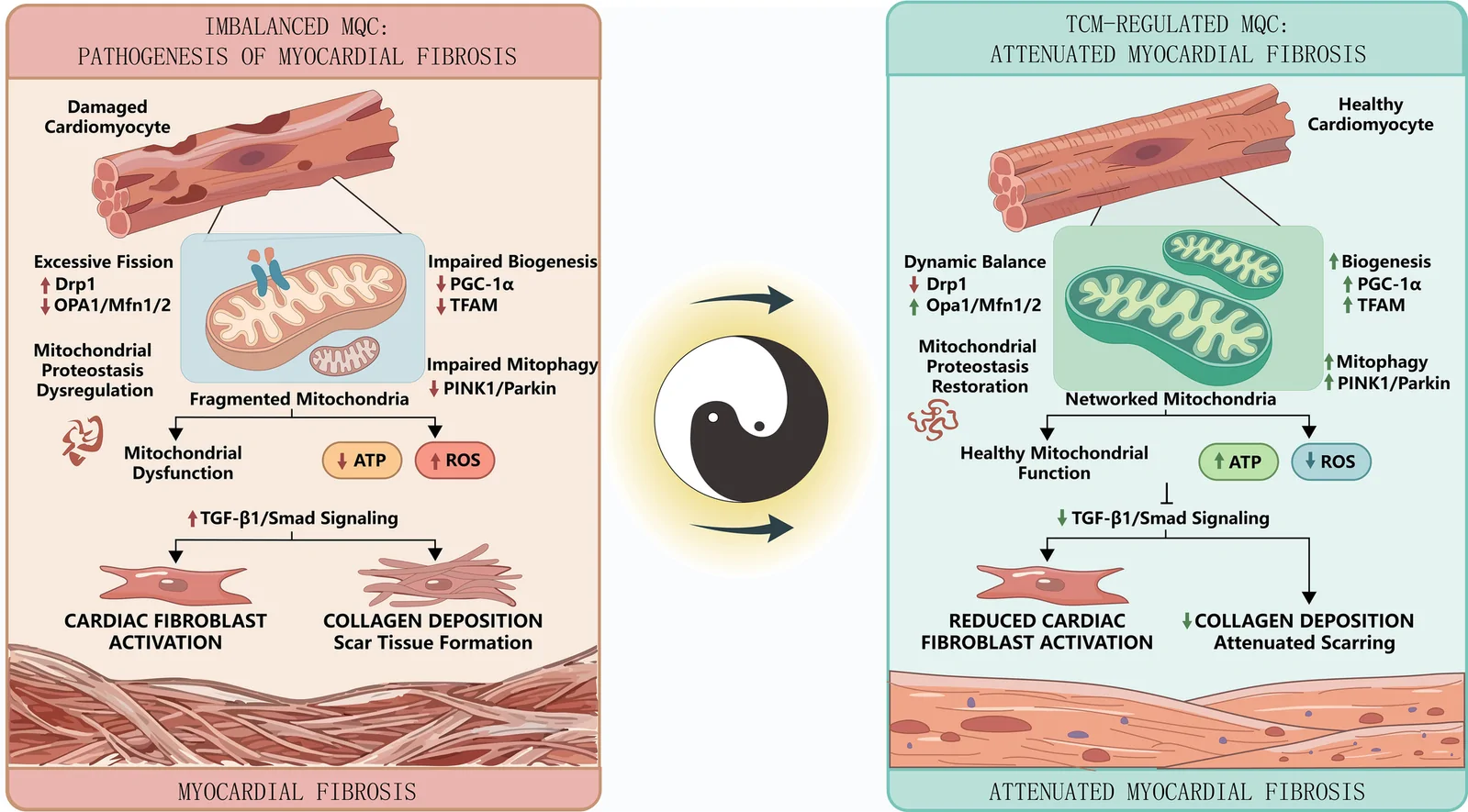
Schematic diagram of mechanisms by which traditional Chinese medicine improves myocardial fibrosis through regulating mitochondrial quality control. This diagram employs a “Yin-Yang balance” design to contrast the pathological mechanisms of MF with the intervention effects of TCM. The left side illustrates the vicious cycle driven by MQC dysfunction leading to MF: imbalance in mitochondrial fission/fusion, impaired biogenesis, defective autophagy clearance, and disrupted proteostasis collectively cause mitochondrial dysfunction, thereby activating cardiac fibroblasts, promoting excessive collagen deposition, and ultimately leading to myocardial scarring. The right side depicts the virtuous cycle in which TCM restores myocardial homeostasis via multi-targeted regulation of the MQC network: active ingredients and herbal formulas in TCM can rebalance mitochondrial dynamics, enhance biogenesis, activate autophagy, and improve proteostasis. These actions synergistically ameliorate mitochondrial function, inhibit pro-fibrotic signaling pathways, reduce cardiac fibroblast activation, collagen deposition, and scar formation, thereby ultimately alleviating myocardial fibrosis.

Consistent with this evidence stratification, the mechanistic statements in this review should be interpreted in the context of evidence strength. While changes in MQC-associated protein expression are frequently reported, we preferentially discuss studies that include functional readouts (e.g., assessing mitophagy activity or mitochondrial homeostasis-related functional outcomes) and/or causal perturbation (genetic gain/loss-of-function) when available. Where studies mainly relied on static protein markers, we highlight the limitation and avoid over-interpreting correlation as causality.

### 3.1 TCM in improving mitochondrial biogenesis dysregulation

#### 3.1.1 Monotherapeutic strategies of Chinese herbal monomers targeting mitochondrial biogenesis

Mitochondrial biogenesis is a key process in maintaining normal cardiac structure and function and in alleviating MF and oxidative stress. Resveratrol (RSV), widely found in plants such as blueberries (*Vaccinium uliginosum L.*) and Japanese knotweed (*Reynoutria japonica Houtt.*), acts as a natural antioxidant that can improve mitochondrial function and promote mitochondrial biogenesis by activating the SIRT1/PGC-1α pathway (86, 87) This compound can also downregulate atrial natriuretic peptide (ANP) and upregulate key factors such as PGC-1α and TFAM (88), modulating SIRT1 to regulate the expression of forkhead box protein 1 (FoxO1) and manganese superoxide dismutase (MnSOD) (89), activating the AMPK pathway, and restoring levels of mitochondrial OXPHOS-related complexes (90), thereby alleviating oxidative stress and optimizing myocardial mitochondrial function in terms of energy metabolism and substrate utilization, ultimately inhibiting MF and delaying the process of cardiac remodeling. Ginsenoside Rd (Rd) is an active compound found in (*Panax Ginseng C. A. Mey.*) that can be absorbed by adipose tissue. Studies have shown that Rd promotes the secretion of membrane proteins from adipocytes via the TANK-binding kinase 1 (TBK1)/AMPK pathway, thereby improving mitochondrial biogenesis via the Wnt family member 5A (WNT5A)/Ca^2+^ signaling pathway, thus alleviating myocardial injury (91). Salidroside (Sal), extracted from *Rhodiola rosea L.*, can, by promoting the AMPK/SIRT1 pathway, upregulate the expression of PGC-1α. NRF1/2, mitochondrial oxidative phosphorylation complex subunits, and iron-sulfur cluster-associated proteins reduce reactive ROS production, stabilize the mitochondrial membrane potential, significantly enhance mitochondrial quality, and maintain normal mitochondrial function (92, 93). Formononetin (FM), an active compound found in *Astragalus membranaceus (Fisch.) Bunge*, is capable of alleviating isoprenaline (ISO)-induced myocardial damage. It acts directly on cardiomyocytes to regulate aldehyde dehydrogenase 2 (ALDH2), hydroxyacyl-coenzyme A dehydrogenase (HADH), and monoamine oxidase B (MAOB), thereby correcting mitochondrial dysfunction and alleviating MF caused by excessive β-adrenergic activation (94). Icariin (ICA), a flavonoid monomer extracted from Epimedium (*Epimrdii Herba*), can alleviate mitochondrial dysfunction by activating the Apelin (APLN)/SIRT3 signaling pathway, thereby upregulating Mfn2, PGC-1α, and the mitochondrial cytochrome b gene (mt-Cyt-b), thereby reducing the accumulation of collagen in cardiac tissue (95). It also enhances NRF2 expression, upregulating heme oxygenase 1 (HO-1), superoxide dismutase 2 (SOD2), and mitochondrial outer membrane translocator 20 (TOM20), thereby inhibiting the accumulation of reactive ROS and protecting the myocardium (96). The natural polyphenol curcumin (Cur) exerts its anti-MF effects by interacting with the PPAR-γ and TGF-β/Smad2/3 signaling pathways to inhibit the production of connective tissue growth factor (CTGF), plasminogen activator inhibitor-1 (PAI-1), and ECM in CFs (97). Collectively, these studies highlight the central role of mitochondrial biogenesis in MF and provide potential targeted intervention strategies for its treatment (Table 2).

**Table 2:** Chinese herbal monomers and active components targeting mitochondrial biogenesis for treating MF.

TCM compounds/Active ingredients	Category	Subject	Test parameters	References
Resveratrol	Polyphenols	dTGR rats, Sprague–Dawley rats, C57BL/6J mice, KKAy mice, neonatal rat cardiomyocytes isolated from the hearts of Wistar rat embryos, mouse embryonic fibroblasts (MEFs) and C2C12 mouse myoblasts	ANP mRNA↓, PGC-1α↑, TFAM↑, NRF1↑, SIRT1↑, cox4↑, MnSOD↑, Ac-FoxO1↓, AMPK↑	(86–90)
Ginsenoside Rd	Tetracyclic triterpenoids	male ICR mice, C57BL/6J mice, H9c2 rat cardiomyocytes, 3T3-L1 mouse preadipocytes, primary neonatal rat ventricular myocytes (NRVMs)	WNT5A↓, p-CAMKII/CAMKII↓, PGC-1α↑, TFAM↑, NRF1↑, NRF2↑	(91)
Salidroside	Glycosides	C57BL/6J mice, H9c2 rat cardiomyocytes, neonatal Sprague-Dawley (SD) rat atrial fibroblasts	PGC-1α↑, OXPHOS↑, NRF1↑, NRF2↑, ROS↓, Fe^2+^↓	(92, 93)
Formononetin	Isoflavones	C57BL/6J mice, HL-1 cardiomyocytes, neonatal SD rat cardiomyocytes	ALDH2↑, HADH↑, MAOB↓, PGC-1α↑, TGF-β1↓, α-SMA↓, COL1A1↓, COL3A1↓, ACTA2↓, TGF-β1↓	(94)
Icariin	Flavonoids	db/db mice and db/+ mice, C57BL/6J mice, primary ventricular cardiomyocytes from newborn (1-day-old) C57 mice	PGC-1α↑, Mfn2↑, mt-Cyt-b↑, Apelin↑, SIRT3↑, NRF2↑, HO-1↑, SOD2↑, TOM20↑, ROS↓	(95, 96)
Curcumin	Polyphenols	spontaneously hypertensive rats (SHRs), Wistar Kyoto (WKY) rats, Rat cardiac fibroblasts (CFs)	PPAR-γ↑, TGF-β1↓, p-Smad2/3↓, CTGF↓, PAI-1↓, COL3↓, FN↓	(97)

#### 3.1.2 Therapeutic strategies of herbal formulas targeting mitochondrial biogenesis for treating MF

A multi-level study on Linggui Zhugan Decoction (LGZGD) predicted that its core mechanism involves B-cell translocation gene 2 (BTG2), manifested as a reduction in the ratios of p-AMPK/AMPK and p-FoxO3a/FoxO3a, and was ultimately validated through molecular docking and kinetic simulations, demonstrating that LGZGD can effectively improve myocardial mitochondrial damage, restore energy metabolism, and protect normal cardiac function (98). In addition, Xinfuli granules (XG) have been shown to increase PGC-1α expression, enhance the ATP/ADP ratio, protect mitochondrial structure and function, and reverse energy metabolism disorders, thereby alleviating the severity of MF (99). Danggui Buxue Decoction (DBD), formulated from a 5:1 ratio of *Astragalus membranaceus (Fisch.) Bunge* and *Angelica sinensis (Oliv.) Diels*, has been shown to enhance the orderliness of myocardial fiber arrangement, decrease collagen fiber accumulation, activate the AMPK/PGC-1α signaling pathway, promote mitochondrial biogenesis, mitigate mitochondrial damage, elevate ATP levels, increase ATP5D protein expression, and improve cardiac function (100). These studies indicate that various TCM formulations can alleviate MF and provide cardioprotective effects by regulating essential signaling pathways, such as AMPK/PGC-1α, thereby enhancing mitochondrial biogenesis and optimizing energy metabolism. This implies that the regulation of mitochondrial function, with the AMPK/PGC-1α axis as a central component, may represent a shared mechanism through which TCM intervenes in MF through multiple tragets (Table 3).

**Table 3:** Herbal formulas targeting mitochondrial biogenesis for treating MF.

Formulation	Efficacy	Composition	Experimental parameters	References
Linggui Zhugan Decoction	Warms the yang, strengthens the spleen and promotes diuresis	*Poria Cocos(Schw.) Wolf.*, *Cinnamomi Ramulus*, *Atractylodes Macrocephala Koidz.*, *Glycyrrhiza uralensis Fisch.*	BTG2↓, p-AMPK↑, p-FoxO3a↑, GSH↑, SOD↑, MDA↓, IL-1β↓, IL-6↓, TNF-α↓	(98)
Xinfuli granule	Nourishes vital energy, promotes blood circulation, dispels dampness and promotes diuresis	*Astragalus membranaceus (Fisch.) Bunge*, *Panax Ginseng C. A. Mey.*, *Salvia miltiorrhiza Bunge*, *Poria Cocos(Schw.) Wolf.*, *Ophiopogon japonicus (L. f.) Ker Gawl.*	PGC-1α↑, ATP/ADP↑, Bcl-2/Bax↑, ATF4↓, CHOP↓, p-eIF2α↓, p-PERK↓, RHOA/ROCK Pathway↓	(99)
Danggui Buxue decoction	Tonify qi and promote blood circulation	*Astragalus membranaceus (Fisch.) Bunge*, *Angelica sinensis (Oliv.) Diels*	p-AMPK/AMPK↑, PGC-1α↑, NRF1↑, mtTFA↑, GLUT4↑, CPT-1↑, ATP5D↑, ATP↑	(100)

### 3.2 TCM improves mitochondrial autophagy regulatory imbalance

#### 3.2.1 Therapeutic strategies of herbal monomers targeting mitochondrial autophagy

Research indicates that inflammatory responses may contribute to MF by activating mitochondrial autophagy. Aloe-emodin (AE) has been shown to inhibit mtROS production that is mediated by the SH3-domain-containing GRB2-like protein B1 (SH3GLB1). This inhibition subsequently affects the PINK1/Parkin signaling pathway and the downstream receptor, Nuclear Dot Protein 52 (NDP52), thereby alleviating radiation-induced cardiac injury (101). Berberine (BBR), a natural bioactive compound extracted from plants such as *Coptis chinensis Franch*, *Phellodendron amurense Rupr.* can reduce oxidative stress damage and preserve mitochondrial function by activating the SIRT6/AMPK/FoxO3a signaling pathway, enhancing mitochondrial biogenesis, and promoting PINK1/Parkin-mediated mitochondrial autophagy, thereby alleviating MF, reducing the area of fibrosis, and mitigating myocardial damage associated with fibrosis-related markers (102). Luteolin (Lut), derived from *Chrysanthemum* ×* morifolium (Ramat.) Hemsl.*, can counteract the diabetes-induced increase in the expression levels of phosphorylated c-Jun N-terminal kinase (p-JNK), phosphorylated c-Jun (p-c-Jun), and miR-221, thereby enhancing myocardial autophagy and reducing the severity of MF (103). Quercetin (Que) can downregulate miR-223-3p expression, enhance FoxO3 expression, and activate the phagocytic pathway, thereby inhibiting CF proliferation, improving myocardial fibrosis, and accelerating myocardial remodeling (104). Ginsenoside Rb1 (Rb1), derived from the traditional Chinese medicinal herb *Panax ginseng C. A. Mey.*, is known for its benefits in treating various cardiovascular diseases. It alleviates Dox-induced myocardial mitochondrial damage through the AMPK/mechanistic target of rapamycin (mTOR) axis. Rb1 reduces p-AMPK upregulation and p-mTOR downregulation, increases NRF2 levels, and decreases Sequestosome-1 (p62/SQSTM1) and LC3-II, thereby exerting a cardioprotective effect (105) (Table 4).

**Table 4:** Herbal monomers and active components targeting mitophagy for treating MF.

TCM compounds/Active ingredients	Category	Subject	Test parameters	References
Aloe-emodin	Anthraquinones	Sprague–Dawley rats, H9C2 cells, HEK293T cells	PTGS2↓, SH3GLB1↓, mtROS↓, NDP52↓, LC3↓, p62↓, PINK1↓, Parkin↓	(101)
Berberine	Isoquinoline alkaloids	C57BL/6J mice	SIRT6↑, p-AMPK↑, FoxO3a↑, COL1A1↓, COL3A1↓, TGF-β↓, α-SMA↓, PINK1↑, Parkin↑, PGC-1α↑, NRF1↑, TFAM↑	(102)
Luteolin	Flavonoids	Sprague–Dawley rats	JNK↓, c-Jun↓, miR-221↓, p62↓, LC3-Ⅱ/LC3-I↑	(103)
Quercetin	Flavonoids	Wistar rats (1 week old) cardiac fibroblasts, 293T human embryonic kidney cells	miR-223-3p↓, FoxO3↑, ATG7↑, p62/SQSTM1↑, LC3-Ⅱ/LC3-Ⅰ↑	(104)
Ginsenoside Rb1	Triterpene saponins	C57BL/6J mice	p62↓, LC3-Ⅱ↓, p-AMPK↓, p-mTOR↑, NRF2↑, GPX4↑, FTH1↑	(105)

#### 3.2.2 Therapeutic strategies of herbal formulas targeting mitophagy for treating MF

Li et al. (106), through target prediction based on ingredient composition combined with multi-omics analysis and experimental validation, revealed that the cardioprotective effect of Shenfuyixin Granules (SFYX) is closely associated with its upregulation of SIRT3 and enhancement of mitochondrial autophagy—a quality control process—which helps to improve mitochondrial function and inhibit apoptosis. A decoction of *Astragalus membranaceus (Fisch.) Bunge* and *Salvia miltiorrhiza Bunge* (Huangqi-Danshen decoction, HDD) inhibits the secretion of miR-27a-3p, which negatively regulates AMPKα2, thereby reducing the PINK1/Parkin pathway-mediated mitochondrial autophagy (107). The serum from Yang-xin-xue keli (YXXKL) reverses Dox-induced myocardial loss and fibrosis by upregulating ATP and modulating mitochondrial DNA quality, thereby reducing LC3B and improving cardiac function (108). Xian et al. (109), demonstrated that NXK promotes PINK1/Parkin-mediated mitochondrial autophagy, thereby effectively maintaining mitochondrial homeostasis and energy metabolism, preventing myocardial mitochondrial dysfunction, and reducing MF. The QiShenYiQi pill (QSYQ) can ameliorate MF by promoting the expression of the phosphatidylinositol 3-kinase (PI3K)/Protein Kinase B (Akt)/mTOR pathway and increasing the number of autophagosomes (110). Xin Fu Kang oral liquid (XFK) has the capacity to modulate both the transcriptional and protein expression levels of PINK1, Parkin, p62, and LC3. This modulation results in a reduction of excessive mitochondrial autophagy and protein degradation within the myocardium of rats suffering from advanced heart failure (111). Additionally, XFK can restore mitochondrial autophagy through the action of Nuclear Receptor Subfamily 4 Group A Member 1 (NR4A1), thereby demonstrating an anti-MF effect (112). In an *in vivo* experiment, Sheng Mai Yin (SMY) was shown to enhance resistance to fatigue and hypoxia in mice, whilst also improving cardiac function and reducing fibrosis and inhibiting excessive mitochondrial autophagy (113). Huangqi Shengmai Decoction (HQSMD) alleviates myocardial pathological damage and downregulates the overexpression of PINK1 and Parkin proteins (114). The compound preparation (PRC) composed of the main active ingredients of Shen-Hong Tongluo Decoction (SHTLF)—Rg1 and Sal—activates the SIRT1/3/PGC-1α/NRF2 axis, normalises PINK1/Parkin expression, downregulates Beclin1, upregulates p62, inhibits excessive mitophagy, enhances cellular activity, restores mitochondrial function, and reduces the degree of fibrosis (115). Tongmai Yangxin Pill (TMYX) regulates the PINK1/Parkin signalling pathway via ERα activation, thereby enhancing mitochondrial function in cardiomyocytes, improving mitochondrial autophagy defects, restoring mitochondrial autophagy balance, and restoring ATP production and ΔΨm. It also alleviates ventricular remodelling, whilst TFAM and NRF1 act synergistically on mtDNA replication and transcription, thereby promoting mitochondrial biogenesis (116). Shihu Mixture (SHM) significantly increases the expression levels of SIRT3, PINK1 and Parkin in the myocardial tissue of model rats and raises the ratio of p-FoxO3a to FoxO3a, whilst simultaneously reducing the expression of p62 protein. It reduces the deposition of collagen fibres in the myocardium, promotes the repair of mitochondrial morphology, increases the number of autophagosomes, activates the autophagy process, and maintains the stability of the intracellular environment, thereby improving cardiac function (117). Qiang-xin 1 Formula (QX1) upregulates the expression of PINK1, Parkin and LC3-II/LC3-I via p-CaMKI, similarly activating mitochondrial autophagy, reducing p62 in mitochondrial fragments, decreasing fibrillar disorganisation and inflammation, and alleviating pathological myocardial damage and cell death (118). Ershen Zhenwu Decoction (EZD), on the other hand, reduces the expression levels of autophagy-related proteins PINK1, Parkin, LC3-II/LC3-I and Beclin1, increases the expression of p62, mitigates excessive mitochondrial clearance, restores energy balance, reduces collagen deposition, and significantly improves cardiac function (119). A variety of natural active ingredients derived from traditional Chinese medicine enhance mitophagy and improve mitochondrial function through distinct signaling pathways. This not only deepens the theoretical connotation of TCM from the perspective of mitochondrial homeostasis but also opens up new avenues for developing novel interventional strategies in TCM (Table 5).

**Table 5:** Herbal formulas targeting mitophagy for treating MF.

Formulation	Efficacy	Composition	Experimental parameters	References
Shenfuyixin Granules	Tonifies qi and warms yang; promotes blood circulation and dispels blood stasis	*Panax Ginseng C. A. Mey.*, *Aconiti Lateralis Radix Praeparata*, *Cinnamomi Ramulus*, *Salvia miltiorrhiza Bunge*, *Paeonia lactiflora Pall.*, *Leonurus japonicus Houtt.*, *Alisma plantago-aquatica L.*, *Polyporus Umbellatus(Pers)Fr.*etc.	SIRT3↑, FoxO3↑, Ac-FoxO1↓, p62↓, ATG5↑, ATG7↑, BNIP3↑, LC3B-II↑	(106)
Huangqi-Danshen decoction	Nourishes qi and blood	*Astragalus membranaceus (Fisch.) Bunge*, *Salvia miltiorrhiza Bunge*	miR-27a-3p↓, AMPKα2/PINK1/Parkin↓	(107)
Yang-xin-xue keli	Warms yang and tonifies qi; promotes blood circulation and dispels cold	*Radix Bupleuri*, Oyster Shell, *Angelica sinensis (Oliv.) Diels*, *Chuanxiong Rhizoma*, *Rehmannia glutinosa (Gaertn.) Libosch. ex Fisch. & C.A.Mey.*, *Paeonia lactiflora Pall.*, *Eleutherococcus giraldii (Harms) Nakai*, *Ziziphi Spinosae Semen*, *Ginseng Radix et Rhizoma*, *Polygonatum odoratum (Mill.) Druce*, *Cinnamomum cassia (L.) D. Don*	MAP1L3B/LC3B↓, ROS↓, ATP↑	(108)
NXK	Tonifies qi, promotes blood circulation and promotes diuresis	*Panax Ginseng C. A. Mey.*, *Ilex pubescens Hook. & Arn.*	PINK1↑, Parkin↑, ROS↓, ATP↑	(109)
QiShenYiQi pill	Tonifies qi, promotes blood circulation and dispels blood stasis	*Astragalus membranaceus (Fisch.) Bunge*, *Salvia miltiorrhiza Bunge*, *Panax Notoginseng (Burk.) F. H. Chen Ex C. Chow*, *Dalbergia odorifera T. C. Chen*	Beclin1↑, LC3B↑, p62↓, p-PI3K/PI3K↓, p-Akt/Akt↓, p-mTOR/mTOR↓	(110)
Xin Fu Kang oral liquid	Tonifies heart qi; promotes blood circulation and unblocks the meridians	*Astragalus membranaceus (Fisch.) Bunge*, *Panax Ginseng C. A. Mey.*, *Angelica sinensis (Oliv.) Diels*, *Chuanxiong Rhizoma*, *Salvia miltiorrhiza Bunge*, *Epimrdii Herba*, *Ganoderma*	PINK1↑, Parkin↑, LC3↑, p62↓, NR4A1↓	(111, 112)
Sheng Mai Yin	Tonifies qi and nourishes yin	*Panax Ginseng C. A. Mey.*, *Ophiopogon japonicus (L. f.) Ker Gawl.*, *Schisandra chinensis (Turcz.) Baill.*	PINK1↓, Parkin↓, ALD↓, BUN↓	(113)
Huangqi Shengmai Decoction	Tonifies the middle, boosts qi and promotes fluid production	*Panax Ginseng C. A. Mey.*, *Ophiopogon japonicus (L. f.) Ker Gawl.*, *Schisandra chinensis (Turcz.) Baill.*, *Astragalus membranaceus (Fisch.) Bunge* etc.	PINK1↓, Parkin↓, BUN↓, ALD↓	(114)
PRC	Activates blood circulation and unblocks meridians	*Panax Ginseng C. A. Mey.*, *Rhodiola rosea L.*	SIRT1↑, SIRT3↑, PGC-1α↑, NRF2↑, Beclin 1↓, p62↑, PINK1↓, Parkin↓	(115)
Tongmai Yangxin pill	Tonifies qi and nourishes yin; unblocks meridians and relieves pain	*Rehmannia glutinosa (Gaertn.) Libosch. ex Fisch. & C.A.Mey.*, *Ophiopogon japonicus (L. f.) Ker Gawl.*, *Spatholobus Suberectus Dunn*, *Glycyrrhiza uralensis Fisch.*, *Tripterygium wilfordii Hook. f.*, Ass-hide Glu*e*, *Schisandra chinensis (Turcz.) Baill.*, *Codonopsis pilosula (Franch.) Nannf.*, *Calendula officinalis L.*, *Ziziphi Spinosae Semen*, *Cinnamomum cassia (L.) D. Don*	ERα↑, TFAM↑, NRF1↑, PGC-1α↑, PINK1 mRNA↓, Parkin mRNA↓, Beclin1 mRNA↓, p62 mRNA↓	(116)
*Shihu* Mixture	Nourishes yin, boosts qi and clears heat; activates blood circulation and dispels stasis; relieves depression and eliminates toxic dampness	*Dendrobium nobile Lindl.*, *Astragalus membranaceus (Fisch.) Bunge*, *Schisandra chinensis (Turcz.) Baill.*, *Anemarrhena asphodeloides Bunge*, *Radix Puerariae*, *Salvia miltiorrhiza Bunge*, *Coptis chinensis Franch*, Earthworm	SIRT3↑, PINK1↑, Parkin↑, LC3↑, p62↓, p-FoxO3a/FoxO3a↑	(117)
Qiang-xin 1 Formula	Boosts qi and promotes fluid production, tonifies and clears the heart	*Persicaria orientalis (L.) Spach*, *Astragalus membranaceus (Fisch.) Bunge*, *Poria Cocos(Schw.) Wolf.*, *Salvia miltiorrhiza Bunge*, *Schisandra chinensis (Turcz.) Baill.*	PINK1↑, Parkin↑, LC3-II/LC3-I↑, p62↓, p-CaMKI↑	(118)
Ershen Zhenwu Decoction	Tonifies qi, warms yang, activates blood circulation and promotes diuresis	*Aconiti Lateralis Radix Praeparata*, *Ginsen Radix Et Rhizoma Rubra*, *Salvia miltiorrhiza Bunge*, *Poria Cocos(Schw.) Wolf.*, *Atractylodes Macrocephala Koidz*, *Paeonia lactiflora Pall.*, *Zingiber Officinale Roscoe*	PINK1↓, Parkin↓, LC3-II/LC3-I↓, Beclin1↓, p62↑	(119)

### 3.3 TCM improves mitochondrial dynamics dysregulation

#### 3.3.1 Therapeutic strategies of herbal monomers targeting mitochondrial dynamics

Rhein (RH) can attenuate MF levels, reduce the cross-sectional area of cardiomyocytes, and decrease the p-Drp1 S616/Drp1 ratio. Additionally, it lowers the expression of cysteine-dependent aspartate-specific protease 9 (caspase 9), cleaved caspase-3, and Bcl-2-associated X protein (Bax), while simultaneously increasing the expression levels of OPA1, Mfn1, and Mfn2 (120). Studies have confirmed that Pae can also restore mitochondrial fusion via the Protein Kinase C Epsilon (PKCε)/Signal Transducer and Activator of Transcription 3 (Stat3)/Mfn2 pathway, thereby alleviating the extent of damage caused by Dox-induced myocardial fibrosis (MF) and cardiac atrophy (121). Baicalein (BE) reduces ISO-induced oxidative stress and MF by regulating the balance between mitochondrial fusion and fission and by inhibiting the Glucose-Regulated Protein 78 (GRP78)/C/EBP-Homologous Protein (CHOP) pathway (122). Breviscapine (BRE) can, as described above, target FoxO3a/Mfn1 regulated mitochondrial fusion, reduce the phosphorylation level of FoxO3a, promote Mfn1 expression, reduce mitochondrial damage, and alleviate fibrotic remodelling (123). Punicalagin (PG) exerts its anti-MF effects by interacting with and inhibiting PTP1B, thereby increasing Stat3 phosphorylation and enhancing the transcriptional expression of OPA1 (124) (Table 6).

**Table 6:** Herbal monomers and active components targeting mitochondrial dynamics for treating MF.

TCM compounds/Active ingredients	Category	Subject	Test parameters	References
Rhein	Anthraquinones	C57BL/6J mice, neonatal rat cardiomyocytes (NRCMs) isolated from 1 to 3-day-old Sprague-Dawley rats, and H9c2 cells.	p-Drp1 S616/Drp1↓, OPA1↑, Mfn1↑, Mfn2↑, caspase 9↓, cleaved-caspase 3↓, Bax↓, Bcl-2↑, ANP↓, BNP↓, β-MHC↓	(120)
Pae	Phenols	Sprague-Dawley rats, Primary cardiomyocytes from Sprague-Dawley rats.	PKCε↑, p-Stat3↑, Mfn2↑	(121)
Baicalein	Flavonoids	BALB/c mice and HL-1 cells.	Caspase 3↓, caspase 9↓, cleaved PARP↓, Drp1↓, MFF↓, GRP78↓, p-PERK↓, ATF4↓, CHOP↓	(122)
Breviscapine	Flavonoids	C57BL/6J mice, neonatal rat cardiomyocytes (NRCMs) and cardiac fibroblasts (NRCFs) from Sprague-Dawley rats, H9c2 cardiomyocytes, and MCF-7 cells.	p-FoxO3a↓, Mfn1↑, NQO1↑, SOD2↑, CAT↑, mtROS↓,	(123)
Punicalagin	Polyphenols	Sprague-Dawley rats and primary cardiomyocytes from Sprague-Dawley rats.	PTP1B↓, p-Stat3↑, OPA1↑	(124)

#### 3.3.2 Therapeutic strategies of herbal medicine pairs targeting mitochondrial dynamics

The drug combination of Astragali Radix and Notoginseng Radix et Rhizoma can maintain mitochondrial homeostasis by reducing p-Drp1 levels, increasing the expression of Mfn2 and OPA1, and improving the processes of mitochondrial fusion and fission (125).

#### 3.3.3 Therapeutic strategies of herbal formulas targeting mitochondrial dynamics

In terms of compound research, SGI, the active ingredient complex of Sini Decoction (SND), can regulate mitochondrial energy metabolism and dynamics in Dox-damaged H9c2 cells, thereby alleviating MF and improving symptoms of cardiomyopathy (126). QSYQ can also downregulate key mitochondrial proteins, such as the mitochondrial Ca^2+^ uniporter (MCU), membrane-associated RING-CH-type finger 5 (MARCHF5) and mitochondrial fission process 1 (MTFP1), thereby inhibiting Drp1-mediated excessive fission. This, in turn, improves myocardial energy metabolism and cell survival, exerting a cardioprotective effect (127). Fufang Zhenzhu Tiaozhi (FTZ) ameliorates MF in diabetic mice by modulating abnormal cardiac lipid metabolism and mitochondrial dynamics (128). Furthermore, Wang et al. (129), demonstrated that Ginseng Dingzhi Decoction (GN) protects cardiomyocytes by increasing mitochondrial phagocytosis, inhibiting mitochondrial fragmentation and enhancing mitochondrial biosynthesis. It acts via the transmembrane BAX inhibitor-1 motif-containing protein 6 (TMBIM6) protein pathway, thereby maintaining mitochondrial function and endoplasmic reticulum homeostasis and alleviating myocardial injury and MF. Qishentaohong granules (QSTH) can restore the balance between mitochondrial fission and autophagy, providing new evidence for the integrated traditional Chinese and Western medicine treatment of MF (130). Additionally, Xin-Ji-Er-Kang (XJEK), a formulation composed of fourteen herbs, promotes the nuclear translocation of nuclear receptor subfamily 3 group C member 1 (NR3C1), upregulates Mfn2 expression, and inhibits the release of mtDNA, thereby suppressing the consequent overactivation of the cGAS/STING signalling pathway and downstream inflammatory responses. This ameliorates MF (131). Furthermore, XJEK treatment alleviates ultrastructural abnormalities in mitochondria, thereby restoring mitochondrial function (132). Guanxin V (GX-V) can inhibit the expression of proteins associated with mitochondrial fission, maintain mitochondrial dynamics homeostasis, and counteract MF (133). The Yiqi Huoxue Formula (YQHX) upregulates retinoic acid receptor-related orphan receptor alpha (RORα), alters the balance of the RORα/Rev-erbα axis promotes mitophagy, rebalances mitochondrial dynamics under pressure overload, and reduces the accumulation of blue-stained collagen fibres, thereby exerting cardioprotective effects (134) (Table 7).

**Table 7:** Compound TCM formulations targeting mitochondrial dynamics for the treatment of MF.

Formulation	Efficacy	Composition	Experimental parameters	References
Sini decoction	Warm the Middle Jiao and dispel cold, restore Yang and rescue from collapse	*Aconiti Lateralis Radix Praeparata*, *Zingiberis Rhizoma*, *Glycyrrhiza uralensis Fisch.*	Sdha↑, Acsl1↑, Ogdh↑, Cpt1b↑, Cpt2↑, Mfn1↑, Mfn2↑, OPA1↑	(126)
QSYQ	Boost Qi and activate blood circulation	*Astragalus membranaceus (Fisch.) Bunge*, *Salvia miltiorrhiza Bunge*, *Panax Notoginseng (Burk.) F. H. Chen Ex C. Chow*, *Dalbergia odorifera T. C. Chen*	MCU↓, MARCHF5↓, MTFP1↓, p-Drp1 Ser616↓, p-Drp1 Ser637↑	(127)
Fufang Zhenzhu Tiaozhi	Functions: Soothe the Liver and fortify the Spleen, activate blood circulation and resolve stasis, and tonify the Liver and Kidney	*Citrus medica “Fingered”*, *Fructus Ligustri Lucidi*, *Salvia miltiorrhiza Bunge*, *Panax Notoginseng (Burk.) F. H. Chen Ex C. Chow*, *Coptis chinensis Franch*, *Atractylodes Macrocephala Koidz*, *Cirsium japonicum Fisch. ex DC.*, *Eucommia ulmoides Oliv.*	Mfn2↑, OPA1↑, Drp1↓, Fis1↓, CD36↓, FABP3↓, CPT1↓	(128)
Ginseng Dingzhi Decoction	Boost Qi and fortify the Spleen, calm the Heart and tranquilize the mind	*Panax Ginseng C. A. Mey.*, *Atractylodes Macrocephala Koidz*, *Poria Cocos(Schw.) Wolf.*, *Dioscorea polystachya Turcz.*, Xylooligosaccharides	PINK/Parkin↑, Drp1↓, Fis1↓, MFF↓, Bax↓, ATG5/ATG12↑, PGC-1α↑, NLRP3↓	(129)
Qishentaohong granules	Boost Qi, activate blood circulation, resolve stasis, and unblock the collaterals	*Codonopsis pilosula (Franch.) Nannf.*, *Astragalus membranaceus (Fisch.) Bunge*, *Persicae Semen*, *Carthamus tinctorius L.*, *Salvia miltiorrhiza Bunge*, *Polyporus Umbellatus(Pers)Fr.*, *Lycopus lucidus Turcz. ex Benth.*, *Mori Cortex*, *Stellaria alsine Grimm*	MFF↓, LC3B↑, FUNDC1↑, BNIP3↑, PARKIN↑, p62↓, p-PINK1 Ser228↑, p-CaMKIIδ T287↓, mito-LC3B/Drp1 S616↑, Drp1↓	(130)
Xin-Ji-Er-Kang	Boost Qi and nourish Yin	*Panax Ginseng C. A. Mey.*, *Ophiopogon japonicus (L. f.) Ker Gawl.*, *Astragalus membranaceus (Fisch.) Bunge*, *Polygonatum odoratum (Mill.) Druce*, *Glycyrrhiza uralensis Fisch.* etc.	Mfn2↑, p-STING↓, p-TBK1↓, TNFα↓, IL-1β↓, cGAS↓, NR3C1↑	(131)
Xin-Ji-Er-Kang	Boost Qi and nourish Yin	*Panax Ginseng C. A. Mey.*, *Ophiopogon japonicus (L. f.) Ker Gawl.*, *Astragalus membranaceus (Fisch.) Bunge*, *Polygonatum odoratum (Mill.) Druce*, *Glycyrrhiza uralensis Fisch.* etc.	SIRT3↑, OPA1↑, Drp1↓, p-Drp1↓, ERα↑, SIRT3↑, Bax↓, Bcl-2↑	(132)
Guanxin V	Boost Qi, nourish Yin, cool the blood, and resolve stasis	*Codonopsis pilosula (Franch.) Nannf.*, *Ophiopogon japonicus (L. f.) Ker Gawl.*, *Schisandra chinensis (Turcz.) Baill.*, *Paeonia lactiflora Pall.*, *Salvia miltiorrhiza Bunge*, *Rehmannia glutinosa (Gaertn.) Libosch. ex Fisch. & C.A.Mey.*	Drp1↓, Fis1↓, TGF-β1↓, NF-κB↓	(133)
Yiqi Huoxue Formula	Tonify Qi and activate blood circulation	*Astragalus membranaceus (Fisch.) Bunge*, *Rhodiola rosea L.*, *Pollen Typhae*, Faeces Trogopterori, *Salvia miltiorrhiza Bunge*, *Angelica sinensis (Oliv.) Diels*, *Carthamus tinctorius L.*, *Codonopsis pilosula (Franch.) Nannf.*	RORα↑, p-Drp1 Ser616↓, Mfn2↑, L-OPA1/S-OPA1↑, PINK1↑, Parkin↑	(134)

### 3.4 TCM in regulating mitochondrial protein homeostasis imbalance

#### 3.4.1 Herbal monomer therapeutic strategies targeting mitochondrial protein homeostasis

Naringin (NAR) can inhibit the phosphorylation of p38 and p53, modulate the mitochondrial apoptosis pathway involving Bcl-2/Bax, downregulate the expression of Bax and Bcl-2 homologous antagonist killer (Bak), elevate Bcl-2 levels, alleviate mitochondrial dysfunction, and inhibit cardiomyocyte apoptosis (135). Furthermore, it can upregulate the levels of heat shock protein 27 (HSP27) and HSP70, stabilise mitochondrial proteins, reduce the degree of MF, and exert cardioprotective effects (136, 137). Schisandrol B (SolB), a representative active ingredient from the Chinese herb *Schisandra chinensis (Turcz.) Baill.*, can reduce excessive iron ion accumulation in the myocardium, inhibit the overactivation of p53, improve mitochondrial function and lipid metabolism, and ameliorate myocardial structural disorganisation and inflammatory infiltration, thereby protecting myocardial tissue (138). Studies have shown that deficiency of the LONP1 protein in cardiomyocytes disrupts the stability of mitochondria-associated endoplasmic reticulum membrane (MAM) contacts, impairs mitochondrial fusion, and activates the endoplasmic reticulum unfolded protein response (ER UPR), ultimately leading to metabolic dysregulation and pathological cardiac remodelling. Artesunate (ART) has been confirmed to directly target LONP1, and research indicates that it can downregulate fibrosis-related genes (139). Therefore, ART may also directly target the activity of this protease, improve the mitochondrial microenvironment, and thereby alleviate MF (140). Ginsenosides (GS), a class of glycosidic compounds, exert cardioprotective effects through multiple pathways. They can modulate the HSP70-p53 interaction to inhibit pulmonary fibrosis (141) and also specifically protect the myocardium: on the one hand, via the TBK1-AMPK pathway and the WNT5A/Ca²⁺ pathway (91). On the other hand, they enhance Akt phosphorylation, thereby inhibiting MF (142), indirectly suppress aberrant translation, promote the clearance of misfolded proteins, reduce fibrotic protein expression, and protect cardiomyocytes from damage. When HSP70 function is impaired, the chaperone-assisted selective autophagy process it mediates is affected. This prevents misfolded or damaged proteins from being effectively recognised and transported to lysosomes for degradation, thereby disrupting protein homeostasis within cardiomyocytes and inducing apoptosis (143). Related to this mechanism, berberine (BBR) can downregulate the expression of GRP78, thereby preventing the accumulation of misfolded proteins, alleviating endoplasmic reticulum stress following myocardial injury, and consequently ameliorating MF (144) (Table 8).

**Table 8:** Herbal monomers and active ingredients targeting mitochondrial protein homeostasis for the treatment of MF.

TCM Compounds/Active Ingredients	Category	Subject	Test parameters	References
Naringin	Flavonoids	H9c2 cells	MMP↑, caspase 3↓, caspase 8↓, caspase 9↓, p-p38↓, p-p53↓, Bax↓, Bak↓, Cyt-c↓, Bcl-2↑, HSP27↑, HSP70↑	(135–137)
Schisandrol B	Lignans	C57BL/6J mice, H9c2 cells	p53↓, SLC7A11↑, GPX4↑	(138)
Artesunate	Sesquiterpene lactones	C57BL/6J mice, iPSCs, iPSC-CFs, human primary fibroblasts, iPSC-CMs, iPSC-ECs, 3D-EHTs	MD2↓, TLR4↓, COL1A1↓, ACTA2↓, CTGF↓, POSTN↓, FN1↓, NPPA↓, NPPB↓, MYH7↓, ACTA1↓, ACTN1↓	(139, 140)
Ginsenoside Rg1	Triterpenoid saponins	Sprague-Dawley rats	HIF-1↑, VEGF↑, p-Akt↑, p38 MAPK↓	(142)
BBR	Isoquinoline alkaloids	Wistar rats	Bcl-2↑, Bax↓, caspase 3↓, GRP78↓, CHOP↓, caspase 12↓	(144)

#### 3.4.2 Herbal formula therapeutic strategies targeting mitochondrial protein homeostasis

Shenxiong Glucose Injection (SGI) can ameliorate G2/M phase arrest, prevent DNA damage when cells enter mitosis, maintain the genomic integrity of progeny cells (145), reduce PARP cleavage, preserve protein homeostasis, inhibit apoptosis, and thereby exert cardioprotective effects. Qihuang Zhuyu Formula (QHZYF), derived from the Buyang Huanwu Decoction, has been previously demonstrated to possess cardioprotective effects. It mitigates cardiomyocyte apoptosis and myocardial hypertrophy by regulating the ubiquitination and degradation of p53, concurrently reducing the degree of MF. This provides a novel perspective for TCM formulas targeting protein stability (146). Zishen Huoxue Decoction (ZSHX) can regulate mitophagy and the UPR^mt^ by modulating the SIRT5/β-tubulin axis. It increases the levels of mitolysosomes and the LC3-II/TOM20 ratio, inhibits the overactivated UPR^mt^ and Drp1 expression, restores the expression of TOM20/Mfn1/Mfn2, enhances mitochondrial biogenesis, promotes mitochondrial network regeneration, and protects cardiomyocytes (147) (Table 9).

**Table 9:** Herbal formulas targeting mitochondrial protein homeostasis for treating MF.

Formulation	Efficacy	Composition	Experimental parameters	References
Shenxiong Glucose Injection	Activate blood circulation, resolve stasis, unblock the Heart, and nourish the Vessels	Ligustrazine Hydrochloride, *Salvia miltiorrhiza Bunge*	cTnT↓, Bcl-2↑, p-ERK1/2↓, Bax↓, Cyt-c↓, caspase 3↓, PARP↓	(145)
Qihuang Zhuyu Formula	Activate blood circulation, resolve stasis, boost Qi, and strengthen the Heart	*Astragalus membranaceus (Fisch.) Bunge*, *Polygonatum sibiricum Redouté*, *Biancaea sappan (L.) Tod.*, *Carthamus tinctorius L.*, Leech	Bmal1↑, Mdm2↑, p53↓	(146)
Zishen Huoxue Decoction	Enrich the Kidney, replenish the Marrow, activate blood circulation, and unblock the collaterals.	*Pleuropterus multiflorus (Thunb.) Nakai*, *Lycium chinense Mill.*, *Alpinia oxyphylla Miq.*, *Mori Fructus*, *Radix Puerariae*, *Salvia miltiorrhiza Bunge*, *Crataegus pinnatifida Bunge*, *Acorus gramineus Soland.*, *Curcuma aromatica Salisb.*, *Polygala tenuifolia Willd.*, Scorpion, *Schisandra chinensis (Turcz.) Baill.*	TOM20↑, ROS↓, MDA↓, MMP↑, Drp1↓, TOM20, Mfn1↑, Mfn2↑, LC3-II↑, Bax↓, caspase 3↓, caspase 9↓	(147)

## 4 Challenges and perspectives

### 4.1 Challenges in translating preclinical findings

Although current preclinical evidence supports the protective role of TCM in MF through MQC regulation, major barriers remain before these findings can be translated into clinical practice. One key issue is the substantial heterogeneity among experimental models. Studies have used different species, cell types, and injury models, each capturing only part of the complex biology of human MF. As a result, therapeutic effects observed in one model may not be reproduced in another. In addition, routes of administration differ widely across studies, including gavage, intraperitoneal injection, and direct treatment in cell culture, which complicates comparison and reduces translational relevance. Dosage selection is another challenge, as many experimental doses are difficult to match in clinical settings. Pharmacokinetic behavior is also highly variable among compounds and formulas, with absorption, metabolism, tissue distribution, and elimination often differing markedly between animals and humans. These factors together limit the direct extrapolation of preclinical results. Moreover, most existing studies focus on short-term biochemical endpoints rather than long-term functional outcomes, making it difficult to determine whether MQC modulation can produce sustained anti-fibrotic benefit. Therefore, more standardized experimental designs and well-structured translational studies are needed to bridge the gap between laboratory evidence and clinical application. Another key challenge is the uneven availability of functional and causal MQC evidence. Many preclinical reports quantify MQC-associated proteins but do not directly demonstrate mitophagy/mitochondrial functional activity with techniques such as mitophagy flux assays or mitochondrial-targeted reporters, nor do they systematically use gain- or loss-of-function approaches (and rescue experiments) to establish MQC causality. Standardized mechanistic designs incorporating functional assays and causal perturbation are therefore recommended to strengthen MQC-directed therapeutic claims in myocardial fibrosis.

### 4.2 Safety, herb-drug interactions, and toxicity

Safety is a central concern for the clinical development of TCM-based interventions. Although many TCM monomers and formulas appear well tolerated in experimental studies, their safety profiles are often incompletely characterized, especially under long-term use or in patients with multiple comorbidities. The multicomponent nature of TCM also increases the complexity of toxicity evaluation, because interactions among ingredients may alter efficacy or produce unexpected adverse effects. In clinical settings, herb-drug interactions are particularly important for patients with cardiovascular disease, who often receive multiple medications simultaneously. TCM may affect drug metabolism, transport, or pharmacodynamic responses, potentially changing the effectiveness or safety of standard cardiac therapies. In addition, some herbs or herbal combinations may carry intrinsic toxicity risks depending on preparation method, dosage, or treatment duration. These concerns are amplified by the lack of systematic toxicological data for many formulations. Consequently, future studies should not only assess anti-fibrotic efficacy, but also examine organ toxicity, dose tolerance, chronic exposure, and potential interactions with conventional drugs. A more rigorous safety evaluation framework will be essential for moving promising TCM interventions from experimental use to clinical practice.

### 4.3 Standardization and quality control of TCM formulas

Standardization remains one of the most difficult obstacles to the clinical translation of TCM, especially for multi-herb formulas. Unlike single-compound drugs, TCM prescriptions contain multiple active constituents with variable chemical stability, bioavailability, and pharmacological contributions. Their composition can be affected by plant source, growth conditions, harvest time, storage, processing, and extraction methods, resulting in substantial batch-to-batch variability. This variability makes it difficult to ensure consistent therapeutic effects across studies or manufacturing lots. Another challenge is that marker compound content alone may not fully represent the biological activity of a formula, since the therapeutic effect often depends on the coordinated action of multiple components. Therefore, quality control must move beyond single-marker quantification toward more comprehensive multi-component evaluation. At present, however, analytical standards for many formulas remain incomplete or inconsistent. This limits reproducibility, hinders regulatory approval, and weakens clinical confidence in TCM products. To improve standardization, future work should establish robust chemical fingerprints, validated quantitative methods, and unified manufacturing specifications. Such efforts will be essential for ensuring product consistency, strengthening evidence quality, and supporting broader clinical adoption of TCM interventions for MF.

### 4.4 Future perspectives

Despite these limitations, MQC-based TCM research still holds considerable promise. Future studies should move toward more rigorous and integrated research strategies that combine mechanistic exploration with translational validation. First, multi-omics approaches and systems biology tools should be used to identify the key components and targets responsible for MQC regulation, helping to clarify which ingredients or combinations drive anti-fibrotic effects. Second, pharmacokinetic and pharmacodynamic studies should be strengthened to define clinically relevant doses, optimize administration routes, and improve bioavailability. Third, safety assessment must be expanded to include chronic toxicity, herb-drug interactions, and patient-specific risk evaluation. Fourth, standardized quality control platforms should be developed for both monomers and complex formulas to ensure reproducibility and consistency. Finally, well-designed clinical studies are needed to determine whether the promising preclinical effects of TCM can be reliably translated into meaningful benefits for patients with MF. By integrating modern analytical technologies with the holistic principles of TCM, future research may establish a more precise, safe, and effective MQC-targeted therapeutic strategy for myocardial fibrosis.

## 5 Conclusion

Based on the MQC theory, this article explores the mechanisms by which TCM intervenes in MF, further confirming that TCM can ameliorate the progression of MF through multi-target regulation of MQC. The specific mechanisms include: (1) activating signalling pathways such as AMPK/SIRT1/PGC-1α to promote mitochondrial biogenesis and correct its dysregulation; (2) upregulating the expression of Mfn2 and OPA1 while inhibiting p-Drp1 to regulate the dynamic balance of mitochondrial fusion and fission; (3) enhancing the expression of proteins such as BNIP3, Beclin1, and Parkin to promote mitophagy, or restoring autophagic regulatory capacity by intervening in the TGF-β/Smad pathway; and (4) inhibiting the phosphorylation of p38 and p53 or modulating HSP70 function to maintain mitochondrial protein homeostasis. With the changes in modern lifestyles, the incidence of MF continues to rise, and it has become a significant cardiac pathological outcome that severely impacts public health. Disruption of the mitochondrial internal environment is one of the key mechanisms in the development and progression of MF. As the core regulatory system for maintaining mitochondrial quantity, morphology, and functional stability, proper MQC function is crucial for myocardial structural integrity. Therefore, in-depth exploration of effective intervention strategies targeting MQC to restore mitochondrial homeostasis has become an important research direction in the prevention and treatment of MF.

## Glossary

Akt: protein kinase B
AMPK: AMP-activated protein kinase
ANP: atrial natriuretic peptide
ATF4: activating transcription factor 4
ATP: adenosine TriPhosphate
Bax: Bcl-2-associated X protein
Bcl-2: B-cell lymphoma 2
Beclin1: Beclin 1 like protein 1
BNIP3: B-cell lymphoma-2 interacting protein 3
CaMK II: calcium/calmodulin-dependent protein kinase II
Caspase: cysteine-dependent aspartate-specific protease
CFs: cardiac fibroblasts
CHOP: C/EBP-homologous protein
COL1: collagen I
COL3: collagen III
CTGF: connective tissue growth factor
Dox: doxorubicin
Drp1: dynamin-related protein1
ERK1/2: extracellular signal-regulated kinase 1/2
ERα: estrogen-related receptor-α
Fis1: fission protein 1
FN: fibronectin
FoxO1: forkhead box protein O1
FoxO3: forkhead box protein O3
FUNDC1: FUN14 domain containing 1
GLUT4↑: glucose transporters
GRP78: glucose-regulated protein 78
HSP70: heat shock protein 70
LC3: microtuble-associated protein light chain 3
LONP1: lon peptidase 1
MDA: malondialdehyde
MF: myocardial fibrosis
MFF: mitochondrial fission factor
Mfn1/2: Mitofusin1/2
MMP: matrix metalloproteinases
MQC: mitochondrial quality control
mtDNA: mitochondrialDNA
mTOR: mechanistic target of rapamycin
NRF1/2: nuclear respiratory factor 1/2
OPA1: optic atrophy protein 1
OXPHOS: oxidative phosphorylation
p53: tumor protein 53
p62: sequestosome-1
PARP: poly ADP-ribosepolymerase
PGC-1α: peroxisome proliferator-activated receptor gamma coactivator-1alpha
PI3K: phosphatidylinositol 3-kinase
PINK1: PTEN-induced kinase 1
RORα: retinoic acid receptor-related orphan receptor alpha
ROS: reactive oxygen species
SIRT: sirtuin
Smad: Sma mothers against decapentaplegic homologue
SOD: superoxide dismutase
Stat3: signal transducer and activator of transcription 3
TBK1: TANK-binding kinase 1
TFAM: transcription factor A
TGF-β1: transforming growth factor β 1
TOM20: mitochondrial outer membrane translocator 20
UPR^mt^: mitochondrial unfolded protein response
UPS: ubiquitin-proteasome system
USP10: ubiquitin-specific processing peptidase 10
WNT5A: Wnt family member 5A
α-SMA: α-smooth muscle actin.
